# Comparative Transcriptome Analysis Identifies Key Regulatory Genes Involved in Anthocyanin Metabolism During Flower Development in *Lycoris radiata*

**DOI:** 10.3389/fpls.2021.761862

**Published:** 2021-12-15

**Authors:** Ning Wang, Xiaochun Shu, Fengjiao Zhang, Weibing Zhuang, Tao Wang, Zhong Wang

**Affiliations:** ^1^Institute of Botany, Jiangsu Province and Chinese Academy of Sciences, Nanjing, China; ^2^Jiangsu Key Laboratory for the Research and Utilization of Plant Resources, Institute of Botany, Jiangsu Province and Chinese Academy of Sciences, Nanjing, China

**Keywords:** *Lycoris radiata*, transcriptome, anthocyanin, structural genes, phytohormone, transcription factors, dihydroflavonol 4-reductase

## Abstract

*Lycoris* is used as a garden flower due to the colorful and its special flowers. Floral coloration of *Lycoris* is a vital trait that is mainly regulated via the anthocyanin biosynthetic pathway. In this study, we performed a comparative transcriptome analysis of *Lycoris radiata* petals at four different flower development stages. A total of 38,798 differentially expressed genes (DEGs) were identified by RNA sequencing, and the correlation between the expression level of the DEGs and the anthocyanin content was explored. The identified DEGs are significantly categorized into ‘flavonoid biosynthesis,’ ‘phenylpropanoid biosynthesis,’ ‘Tropane, piperidine and pyridine alkaloid biosynthesis,’ ‘terpenoid backbone biosynthesis’ and ‘plant hormone signal transduction’ by Kyoto Encyclopedia of Genes and Genomes (KEGG) enrichment analysis. The candidate genes involved in anthocyanin accumulation in *L. radiata* petals during flower development stages were also identified, which included 56 structural genes (especially *LrDFR1* and *LrFLS*) as well as 27 key transcription factor DEGs (such as *C3H*, *GATA*, *MYB*, and *NAC*). In addition, a key structural gene namely *LrDFR1* of anthocyanin biosynthesis pathway was identified as a hub gene in anthocyanin metabolism network. During flower development stages, the expression level of *LrDFR1* was positively correlated with the anthocyanin content. Subcellular localization revealed that LrDFR1 is majorly localized in the nucleus, cytoplasm and cell membrane. Overexpression of *LrDFR1* increased the anthocyanin accumulation in tobacco leaves and *Lycoris* petals, suggesting that *LrDFR1* acts as a positively regulator of anthocyanin biosynthesis. Our results provide new insights for elucidating the function of anthocyanins in *L. radiata* petal coloring during flower development.

## Introduction

Plant pigments, such as anthocyanins, carotenoids and chlorophylls, play important roles in affecting the appearance of flower, fruit and seed coloring (Tanaka et al., 2008; Rebecca et al., 2010; Rosas-Saavedra and Stange, 2016; Cui et al., 2021). As an important group of plant pigments, anthocyanins are water soluble and belong to the family of flavonoids. So far, more than 500 different anthocyanins have been isolated from plants (Francavilla and Joye, 2020). They are highly involved in determining flower, seed, fruit and vegetative tissue colors, ranging from pink through scarlet, purple, and blue (Tanaka et al., 2008; Khoo et al., 2017). There are six species of anthocyanins (namely cyanidin, delphinidin, peonidin, malvidin, pelargonidin, and petunidin) in colorful plants (Tanaka et al., 2008; Castaneda-Ovañdo et al., 2009), of which cyanidin is responsible for red-purple coloration, delphinidin contributes to purple or blue-red, and pelargonidin contributes to red and orange (Khoo et al., 2017). Besides, anthocyanins also play various vital functions in plant biological functions, including disease protection, resisting environmental stresses, and promoting pollination (Lev-Yadun and Gould, 2009; Zhang et al., 2014).

Anthocyanins are synthesized in cytosol, and stored in the vacuole. Studies on several plant species, including *Arabidopsis* (Baudry et al., 2006; Gonzalez et al., 2008; Qi et al., 2011; Xie et al., 2016), agricultural crops (Yang et al., 2019; Dong et al., 2020), fruits (Rahim et al., 2014; Zhou et al., 2015; Jiang et al., 2019; Li C. et al., 2020), vegetable and ornamental plants (Suzuki et al., 2016; Xu et al., 2017; Jin et al., 2018, 2019; Zhu et al., 2019) have revealed that biosynthesis of anthocyanins are controlled by structural and regulatory genes that take part in formation as well as regulation of specific enzymes. The key enzymes including phenylalanine ammonia lyase (PAL), cinnamic acid 4-hydroxylase (C4H), 4-coumarate-CoA ligase (4CL), chalcone synthase (CHS), chalcone isomerase (CHI), flavonone 3-hydroxylase (F3H), dihydroflavonol 4-reductase (DFR), flavonoid 3′-monooxygenase (F3′H), anthocyanin synthase (ANS), as well as UDP-glucose-flavonoid 3-*O*-glucosyltrasnferase (UFGT) are important in anthocyanin biosynthesis (Koes et al., 2005; Li et al., 2018). Among them, DFR catalyzes the conversion of dihydroflavonols to leucoanthocyanidins, which is one of the final stages of anthocyanin biosynthesis (Shimada et al., 2005; Luo et al., 2016). *DRF* gene is responsible for plant pigmentation (Lou et al., 2014), and its mutation has been associated with the loss of anthocyanins as well as proanthocyanidins (Liu H. et al., 2019; Jiang et al., 2020; Lim et al., 2020; Feng et al., 2021). Besides, enhancement or activation of *DFR* gene expression is vital in MYB transcription factor (TF)-based anthocyanin engineering. For example, regulatory roles of MYB TFs in anthocyanin biosynthesis such as Production of Anthocyanin Pigmentation 1 (*PAP1*, a *MYB75* TF), *PeMYB2/11/12*, *PsMYB114L*, *FtMYBF18*, *EsMYB90*, and *FhMYB5* depend on *DFR* expression (Hsu et al., 2015; Li et al., 2019; Zhang et al., 2019; Dong et al., 2020; Qi et al., 2020). *StMYB44* represses anthocyanin accumulation in leaves of tobacco by directly suppressing the activity of the *DFR* promoter (Liu Y. et al., 2019).

Moreover, some other TFs such as the MYB-bHLH-WD (MBW) complex, B-box, bZIP, MYC, NAC, WRKY, bHLH, MADS-box, and WD could also coordinate anthocyanin biosynthesis initiation by binding to the promoter regions of structural genes (Xu et al., 2015; Zhou et al., 2015; An et al., 2017; Lloyd et al., 2017; Lu et al., 2018; Fang et al., 2019; Jiang et al., 2019). For example, *Arabidopsis* bHLH TFs (GL3, TT8, and EGL3) and WD40 repeat protein TTG1 regulate anthocyanin biosynthetic gene expressions (Gonzalez et al., 2008; Gerats and Strommer, 2009; Saito et al., 2013). Similarly, anthocyanin biosynthesis in petunia petal cells is controlled by the MBW complex, comprising subgroups of MYB TF (PhAN2 or PhAN4) and bHLH TF (PhAN1 or PhJAF13), as well as the WD40 regulator PhAN11 (Quattrocchio et al., 2006). Strawberry FaMADS1a played a negative role in anthocyanin accumulation via repressing expression of *FaPAL6*, *FaCHS*, *FaDFR*, and *FaANS* (Lu et al., 2018). Furthermore, apple B-box zinc finger protein MdBBX20 promotes anthocyanin accumulation in response to ultraviolet-B radiation and low temperature (Fang et al., 2019). DhMYB2 was found to interact with DhbHLH1, thereby regulating anthocyanin secretion in *Dendrobium* hybrid petals (Li et al., 2017). Therefore, the regulatory mechanisms of TFs on plant color are diverse. The formation of plant flower color is affected by both structural genes and TFs.

The *Lycoris* species belongs to Amaryllidaceae family, and is a perennial bulb plant native to Northeast Asia, including China, South Korea, and Japan. It consists of about 20 species, of which 15 species and one variety are distributed in China (Zhang et al., 2020). Among them, *Lycoris radiata* is considered ornamentally and medicinally valuable, as the colorful and special flowers have been used for decoration and the bulbs are notable to produce alkaloids with various biological activities (Park et al., 2019, 2021). Anthocyanins are abundant in *Lycoris* flowers and also contribute to their color formation (He et al., 2011; Chun et al., 2013; Yue et al., 2019; Park et al., 2021). For example, four critical anthocyanins, namely cyanidin 3-sophoroside, cyanidin 3-xylosylglucoside, cyanidin 3-sambubioside, and pelargonidin 3-xylosylglucoside in *L. longituba* tepals of different colors have been well identified (He et al., 2011). In *L. radiata* flowers, three anthocyanins (cyanidin 3-diglucoside, cyanidin 3-sambubioside, and cyanidin 3-glucoside) were identified (Chun et al., 2013), and their presence during four flower development stages was confirmed more recently (Park et al., 2021). However, the molecular mechanisms of anthocyanins regulating color formation of *Lycoris* flower remain unclear. Thus, identifying the key genes related to color formation in *Lycoris* flower would provide a more sufficient genetic resource for manipulation of the related pathways to develop new cultivars with specific flower colors.

In recent years, transcriptome sequencing (RNA-seq) was used as a rapid technique to uncover DEGs, biosynthesis pathways, and TFs related to specific traits in plants (He et al., 2020; Li C. et al., 2020). In this study, we reported the changing profile of anthocyanins and gene expression dynamics in *L. radiata* petals at four developmental stages by integrated analyses of the physiology and transcriptome. We further identified modules with co-expressed genes and candidate hub genes for anthocyanin accumulation, and revealed *LrDFR1* acts as a positive regulator involved in anthocyanin biosynthesis. Our results may serve as a reference for understanding the regulation of key genes and transcription processes in color formation in the flowers of this esthetically important *Lycoris*.

## Materials and Methods

### Plant Materials

**FIGURE 1:**
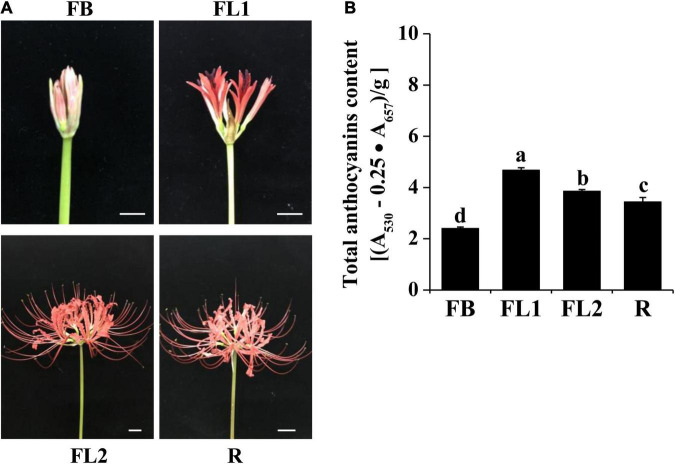
Phenotypes and anthocyanins content in petals of *L. radiata* at different development stages. **(A)** Petals of *L. radiata* at four flower development stages. FB, floral bud stage; FL1, partially opening flower stage; FL2, fully opened flower stage, and R, senescent flower stage. Bars: 1 cm. **(B)** Anthocyanin levels in *L. radiata* petals at four flower development stages. Bars with different letters are significantly different at *p* < 0.05 according to Duncan’s multiple range test.

*Lycoris radiata* (L’Her.) Herb. plants were grown in Experimental Plantation of Institute of Botany, Jiangsu Province and Chinese Academy of Sciences, Nanjing, China. According to the studies reported previously (Yue et al., 2019; Park et al., 2021), three biological replicates of *L. radiata* flowers were sampled at four development stages, which were FB (floral bud stage), FL1 (partially opening flower stage), FL2 (fully opened flower stage) and R (senescent flower stage), as shown in Figure 1A. Each biological replicate was taken from petals of five flowers and pooled together. For gene expression analysis, different *L. radiata* tissues, including scape, stamen, pistil, flower stalk, and petal samples were obtained during flowering time, while leaf, root, as well as bulb samples from the same plants were obtained during the vigorous vegetative growth stage. The fresh samples were harvested and instantly frozen in liquid nitrogen, then kept at –80°C until use.

### Measurement of Total Anthocyanins

Extraction and determination of anthocyanins of *L. radiata* flowers was performed following the protocol of Mehrtens et al. (2005) with minor modifications. Briefly, approximately 0.1 g fresh petals were ground in 1 mL of acidic methanol (0.1 mol L^–1^ HCl) and then incubated overnight in the dark at 4°C with gentle shaking. After centrifugation for 10 min at 12,000 rpm, the supernatant was diluted four times with acidic methanol and the absorbance was measured at 530 and 657 nm using a UV-1600 spectrophotometer (SHIMADZU, Kyoto, Japan). The concentration of anthocyanins was calculated using the following formula: Q_*Anthocyanins*_ = (A_530_ – 0.25 × A_657_) × FW^–1^, where Q_*Anthocyanins*_ is the amount of anthocyanins, A_530_ and A_657_ is the absorption at the indicated wavelengths and FW represents the weight of the fresh sample [g].

### Construction of the cDNA Library, Sequencing, and Transcriptome Assembly

Total RNA was extracted with the mirVana miRNA isolation kit (Thermo Fisher Scientific, Waltham, MA, USA) following the manufacturer’s protocol. The quality and quantity of the RNA were examined by the Agilent 2100 Bioanalyzer (Agilent Technologies, Santa Clara, CA, USA). Samples with RNA Integrity Number (RIN) ≥ 7 were subjected to cDNA library construction using the TruSeq Stranded mRNA LTSample Prep Kit (Illumina, San Diego, CA, United States). Sequencing of the cDNA libraries was done on the Illumina sequencing platform (Illumina HiSeq™ 2500) by Shanghai OE Biotech. Co. Ltd. (Shanghai, China). Reads were cleaned by removing adapters, as well as low-quality and ambiguous regions, then subjected to *de novo* assembly using the Trinity software (Grabherr et al., 2011).

### Functional Annotation

Alignment of the assembled unigenes was done against public databases including National Center for Biotechnology Information (NCBI) non-redundant protein (Nr) and nucleotide (Nt) database, the Swiss-Prot protein database, Gene Ontology (GO) database, Protein Family (Pfam) database, Kyoto Encyclopedia of Genes and Genomes (KEGG) database, Eukaryotic Ortholog Groups (KOG) database, and eggNOG (evolutionary gene genealogy: Non-supervised Orthologous Groups) database.

### Identification of Differentially Expressed Genes

The expression level of unigenes was calculated using fragments per kilobase per million fragments mapped (FPKM) method (Mortazavi et al., 2008). Identification of DEGs among samples at four development stage was done using the DESeq2 package implemented in R software, with cutoff values of |log2 (fold change)| > 1 and *p*-value < 0.05 algorithms (Young et al., 2010). To visualize the differential expression profiles, we generated a heatmap for the Trimmed Mean of *M*-values (TMM) normalized against FPKM via the pheatmap package in R.

### Transcription Factors Analysis

To predict TFs involved in color formation of *L. radiata*, we utilized the getorf database (mini-size 150) to find the open reading frame (ORF) (Rice et al., 2000) and then used the HMM search database (version 3.0) to align the ORFs to the TF protein domain. The aligned sequences were described according to the TF families available on the PlantTF database version 3.0 (Zhang et al., 2011). Moreover, the Pearson’s correlation coefficient (PCC) between these differentially expressed TFs, structure genes and total anthocyanin content of samples was calculated. The TFs with |PCC| > 0.8 were selected for subsequent analysis. The TF expression data, which included expression levels for MYB, bHLH, WD40, and the DEGs identified in the flavonoid biosynthetic pathway, was screened using blastx software, with an *e*-value of 1e-10. The target gene sequence was aligned to the protein sequence of the reference species contained in the string database, and the protein interaction relationship of the reference species was used to construct an interaction network. Network visualization for the interaction network related to DFR and DEGs was performed using Cytoscape version 3.6.1.

### Gene Cloning and Construction of Expression Vectors

Cloning of *LrDFR1* was based on putative ORFs of unigenes from the RNA-seq database. Primers (Supplementary Table 1) were synthesized for ORF sequence amplification using Tks Gflex™ DNA Polymerase (Takara, Dalian, China) from *L. radiata* petal cDNA. Reaction conditions were: 5 min of 95°C, 35 cycles for 30 s at 94°C, 30 s at 60°C, 1 min at 72°C, with extension at 72°C for 10 min. PCR products were cloned into pMD19-T simple vectors (Takara, Dalian, China). Afterward, those T-vectors were transferred into DH5α competent cells (Takara, Dalian, China) for amplification. The overexpression vectors of *LrDFR1* were established by linking their ORFs into a linear plant transformation vector, pBinGFP4, using the One Step Cloning Kit (Vazyme, Nanjing, China). Then the *35S:LrDFR1* recombinant vectors were transformed into *Agrobacterium tumefaciens* EHA105 competent cells.

### Subcellular Localization and Proanthocyanidin Staining

The pBinGFP4 vector with *LrDFR1-GFP* was transformed into the *Agrobacterium tumefaciens* strain EHA105, and transferred into *Nicotiana benthamiana* epidermal cells (Sheludko et al., 2007). Cultivation of the transformed *N. benthamiana* leaves was done for 2–6 days. For co-localization with membrane-localized marker, *35S: PIP2;1*-mCherry construction was used (Huang et al., 2019). Assessment of transformed *N. benthamiana* epidermal cells was observed with confocal laser scanning microscopy (Zeiss LSM780 META, Jena, Germany). For staining of the nuclei, 10 mg/mL 4′6-diamidino-2-phenylindole (DAPI) was infiltrated into *N. benthamiana* leaves 6 h before observation.

Staining of proanthocyanidin was conducted as described by An et al. (2015). Briefly, light-treated *N. benthamiana* leaves were decolorized in a solution of ethanol: glacial acetic acid (3:1). A dimethylaminocinnamaldehyde (DMACA) reagent staining solution (Sigma-Aldrich, St. Louis, MO, United States) was then added for staining.

### Agrobacterium-Mediated Transient Transformation System of *Lycoris* Petals

The *A. tumefaciens* harboring *35S:LrDFR1-GFP* construct and the control pBinGFP4 vector were prepared for injecting into *Lycoris* petals, respectively. The recombinant *Agrobacterium* strains were cultured in YEB broth containing 50 μg mL^–1^ kanamycin and incubated at 28 °C. Then, the collected recombinant *Agrobacterium* strains were resuspended to OD_600_ of 0.6 in a buffer with 10 mM 2-(4-Morpholino) ethanesulfonic acid, 10 mM MgCl_2_, and 120 μM acetosyringone. Transformed *Lycoris* petals were stored for 48 h in the dark after which they were transferred to a phytotron at a constant photon flux density of 100 μmol m^–2^ s^–1^. With 5 days cultivation, *Lycoris* petals were obtained for anthocyanin level assessment and RNA extraction.

### Validation RNA-Seq by Quantitative Real-Time PCR

For validating gene expression using qRT-PCR, 32 unigenes associated with anthocyanin biosynthesis and phytohormone metabolism were randomly selected (Supplementary Table 2). Total RNA isolation was conducted by using the RNAprep Pure Plant Kit (Tiangen, Beijing, China). First-strand cDNA was synthesized with TransScript One-Step gDNA Removal and cDNA Synthesis SuperMix kit (Takara, Dalian, China), and the extracted RNA was used as template according to manufacturer’s instructions. A list of gene-specific primers is provided in Supplementary Table 1. The quantified expression levels of the tested genes were normalized against the house keeping genes *TIP41-like protein* (*TIP41*) according to previous study on *L. aurea* (Ma et al., 2016). qRT-PCR assays were conducted by the SYBR Premix Ex Taq™ II kit (Tli RNaseH Plus) (Takara, Dalian, China) in a Bio-Rad iQ5 Gradient RT-PCR system. Reaction conditions were: 30 s of denaturation at 95°C and 40 amplification cycles (5 s at 95°C, 30 s at 60°C). Calculation of relative target gene expression levels was done using the 2^–ΔΔ*Ct*^ method (Livak and Schmittgen, 2001). Experiments were conducted using three independent biological and three technical replicates.

### Statistical Analysis

Statistical analyses were done by SPSS version 10.0 software (IBM Corporation, Armonk, NY, USA). The significant difference among sets of data was determined by one-way analysis of variance (ANOVA) with Duncan’s multiple range test (*p* < 0.05) or a significant *t*-test (^**^p < 0.01, *p < 0.05). All the results are presented as the mean ± standard deviation (SD).

## Results

### Anthocyanin Levels in *Lycoris radiata* Petal During Flower Development Stages

During the red flower development of *L. radiata*, petals underwent a rapid color change from slight red to brilliant red (Figure 1A). At the flower bud (FB) stage, a slight red color was observed, then the color intensity was significantly increased with rapid elongation of petals in FL1. Subsequently, the intensity of *L. radiata* decreased at FL2 and R stages (Figure 1A). We thus investigated the changes of anthocyanin contents in *L. radiata* at four different petal development stages. Notably, anthocyanin content at FL1 stage was significantly higher than that of FB, FL2 and R stages (Figure 1B), suggesting that changes in anthocyanin levels could be the main reason for red color formation of *L. radiata*.

### Transcriptome Sequencing and *de novo* Assembly

To further study the molecular mechanism of *L. radiata* petal coloring during flower development, twelve libraries were established using samples at four flower development (FB, FL1, FL2, and R) stages (three biological replicates for samples at each development stage), and a total of 644.93 million raw reads as well as 96.73 Gb raw bases were obtained. After eliminating the adaptor, poor-quality sequences, and ambiguous reads, 634.09 million clean reads and 89.86 Gb clean bases were retrieved from 12 samples (Supplementary Table 3). The quality score above 30 (Q30) of each library was 93.75–94.91%, and GC percentages ranged from 44.99–46.51% (Supplementary Table 3). By using Trinity software, the *de novo* assembly of 12 petal transcriptomes totally generated 87,584 unigenes with an average length of 942 bp (Supplementary Table 4). Sequence length distribution showed that 27,073 (30.91%) unigenes had a mean length ≥ 1000 bp (Supplementary Figure 1 and Supplementary Table 4). The N50 was determined to be 1,334 bp, which indicated that the quality of sequence assembly was good.

FPKM values were used to estimate the transcription levels of unigenes. More than 50.0% of unigenes had FPKM values above 1 (Supplementary Figure 2). In addition, the use of relative unigene expression obtained from FPKM for principal component analysis (PCA) showed 52.10% variability among the samples (Supplementary Figure 3). Moreover, heatmap coefficient matrix analysis of the samples based on the FPKM values showed that most biological replicates (except FB3 sample) exhibited similar expression patterns, indicating relatively high reliability of our sequencing data (Supplementary Figure 4).

### Functional Annotations and Unigene Classifications

All of the unigenes were annotated by BLAST search against the public databases. The results revealed that 40,974 (46.78%), 29,476 (33.65%), 37,487 (42.8%), and 22,318 (25.48%) unigenes were annotated to the Nr, Swiss-Prot, eggNOG, and Pfam databases, respectively. Taken the entire public databases together, a total of 41,534 (47.42%) unigenes could be successfully annotated (Supplementary Table 5). To elucidate their main biological functions, GO, KOG, and KEGG pathway assessments were also performed (Supplementary Figures 5–7). Consequently, 27,296 (31.17%) unigenes were assigned into three main categories including “biological process” (BP), “cellular component” (CC), and “molecular function (MF),” which could be further distributed under 50 GO terms (Supplementary Figure 5). In addition. 15,122 (17.27%) unigenes were associated with 126 KEGG pathways, and category ‘Metabolism’ (6187 unigenes) was the most abundant (Supplementary Figure 6). Moreover, the KOG analysis showed that 23,858 (27.24%) annotated unigenes were assigned into 25 classes (Supplementary Figure 7).

### Identification of Differentially Expressed Genes in *Lycoris radiata* Petal During Flower Development Stages

**FIGURE 2:**
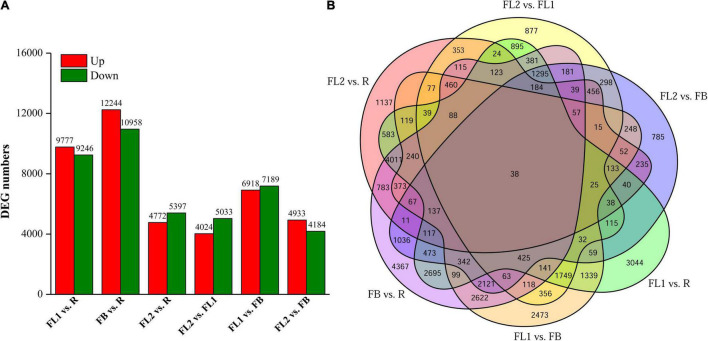
Statistics of differentially expressed genes (DEGs) between two different samples at flower development stages. **(A)** Numbers of DEGs in various pair-wise comparisons. **(B)** Venn diagram for the numbers of DEGs as shown by pair-wise comparisons. FB, floral bud stage; FL1, partially opening flower stage; FL2, fully opened flower stage, and R, senescent flower stage.

To identify the key DEGs involved in *L. radiata* petal color transitions, six pair-wise comparison groups (FL1 vs. R, FB vs. R, FL2 vs. R, FL2 vs. FL1, FL1 vs. FB, and FL2 vs. FB) were conducted (Figure 2). A total of 38,798 DEGs were identified among all samples based on a |log_2_ fold change| >1 at *p* < 0.05. Among these comparison groups, the largest abundance of DEGs (23,202) was found between FB and R libraries, of which 10,958 and 12,244 genes were down-regulated and up-regulated, respectively (Figure 2A). Conversely, the smallest abundance of DEGs (9,057) was recorded between FL2 and FL1 libraries, with 5,033 and 4,024 of them down-regulated and up-regulated, respectively (Figure 2A). Furthermore, the overlap DEGs among the six comparison groups were screened. The results indicated that 38 genes were differentially expressed among all the comparisons, which indicated that these DEGs might have key functions in the color expression of different petals (Figure 2B and Supplementary Table 6).

### Functional Annotation of Differentially Expressed Genes

**FIGURE 3:**
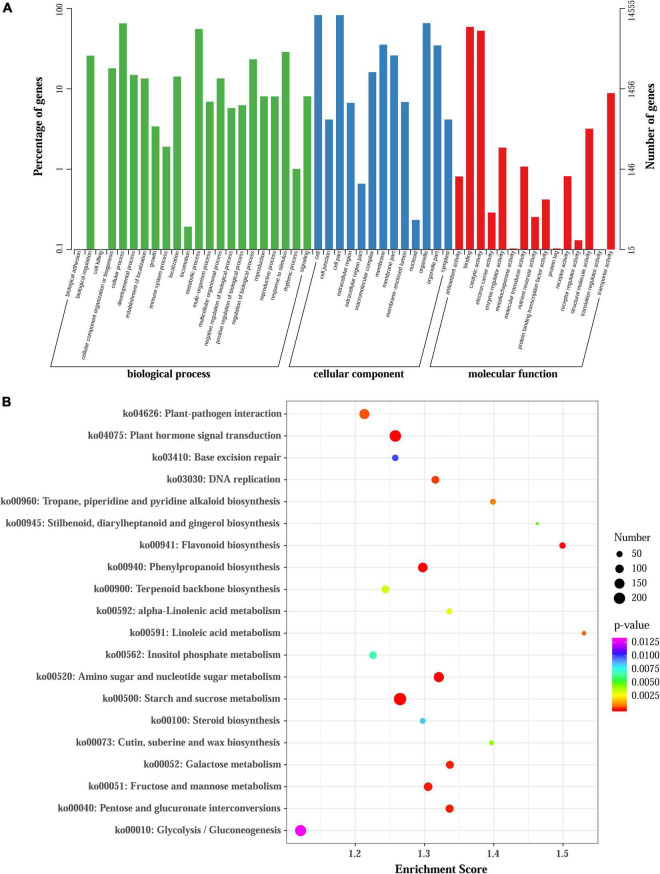
GO and KEGG enrichment analysis of all DEGs. **(A)** GO enrichment results of all DEGs. **(B)** Enrichment of the top 20 KEGG pathways of all DEGs according to the *p*-value.

To elaborate the functions of DEGs and identify genes involved in regulating anthocyanin accumulation in *L. radiata*, all the DEGs were firstly subjected to GO analyses, and 14,555 of the 38,798 DEGs were assigned to GO annotations (Supplementary Table 7 and Figure 3A). In the biological process category, most of the DEGs were mapped to ‘cellular process’ (9,476, 20.23%), ‘metabolic process’ (8,073, 17.23%), and ‘response to stimulus’ (4,181, 8.92%) terms. In the cellular component category, more than 63.08% of DEGs were enriched in ‘cell,’ ‘cell part’ and ‘organelle’ terms, but for molecular function, nearly 86.13% of DEGs were mapped to ‘catalytic activity’ and ‘binding’ terms (Figure 3A). For the KEGG annotation results, 7,631 DEGs among all samples were also mapped to 126 KEGG pathways (Supplementary Table 7). Comparisons across the samples at four petal development stages revealed significant enrichment of DEGs in ‘flavonoid biosynthesis,’ ‘phenylpropanoid biosynthesis,’ ‘Tropane, piperidine and pyridine alkaloid biosynthesis,’ ‘terpenoid backbone biosynthesis’ as well as ‘plant hormone signal transduction’ pathways (Figure 3B and Supplementary Figure 8). For example, the significantly enriched KEGG pathway term ‘Tropane, piperidine and pyridine alkaloid biosynthesis’ was shared in all the comparisons. The ‘flavonoid biosynthesis’ pathway was enriched in FL1 vs. R, FB vs. R, FL2 vs. R, FL2 vs. FL1, and FL2 vs. FB, but not in FL1 vs. FB. In addition, the ‘plant hormone signal transduction’ pathway was enriched in FB vs. R, FL2 vs. FB, and FL2 vs. FL1 (Supplementary Figure 8).

### Identification of Key Differentially Expressed Genes Responsible for the Anthocyanin Biosynthesis Pathway

**FIGURE 4:**
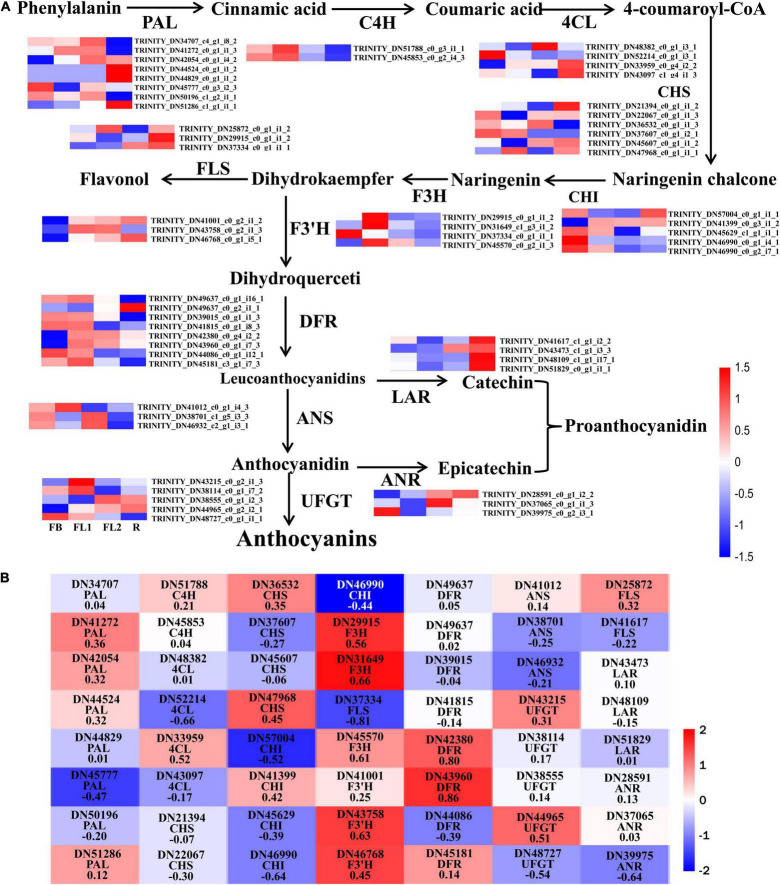
Analysis of DEGs involved in anthocyanin biosynthesis pathway in *L. radiata.* **(A)** Anthocyanin biosynthesis pathway and the log_2_ transformed FPKM values of DEGs associated with structural enzyme genes were used to draw the heatmap. The enzymes include 4-coumarateCoA ligase (4CL), phenylalanine ammonia lyase (PAL), chalcone synthase (CHS), flavone 3-hydroxylase (F3H), chalcone isomerase (CHI), flavonoid 3′-hydroxylase (F3′H), dihydroflavonol reductase (DFR), flavonol synthase (FLS), UDP-flavonoid glucosyl transferase (UFGT), anthocyanidin reductase (ANR), and leucoanthocyanidin reductase (LAR). FB, floral bud stage; FL1, partially opening flower stage; FL2, fully opened flower stage, and R, senescent flower stage. Color gradients comprise red, white, and blue, representing genes that were upregulated, not regulated, as well as downregulated, respectively. **(B)** The heatmap analysis of all DEGs in anthocyanin biosynthesis pathway according to the FPKM value.

To elucidate the molecular basis underlying difference in anthocyanin biosynthesis among the four flower development stages in *L. radiata*, DEGs involved in the anthocyanin synthesis pathway were identified. The results revealed that 56 DEGs were enriched in the anthocyanin synthesis pathway, including *PAL*, *C4H*, *4CL*, *CHS*, *CHI*, *F3H*, *F3′H*, *DFR*, *ANS*, *UFGT*, *FLS*, *ANR*, and *LAR* (Figure 4A). Moreover, the Pearson’s correlation coefficient between the expression level of these DEGs and the total anthocyanins content was further calculated (Figure 4B). The results showed that 23 DEGs negatively regulated anthocyanin synthesis, whereas 33 DEGs positively regulated the anthocyanin synthesis. Among them, the expression level of two DEGs, namely *LrDFR1* (DN43960) and *LrDFR2* (DN42380) indicated a significant positive correlation with the total anthocyanins content in petals during the flower development stages, while *LrFLS* (DN37334) indicated a significant negative correlation with the total anthocyanins content (|PCC| > 0.8, Figure 4B and Table 1), suggesting that these three DEGs may have an essential role in anthocyanin accumulation.

**TABLE 1:** The candidate TFs and the key structural gene involved in anthocyanin accumulation.

Gene family	Gene ID	Annotation	Correlation with total anthocyanin	*p*-value
*FLS*	TRINITY_DN37334_c0_g1_i1_1	Flavonol synthase/flavanone 3-hydroxylase-like	−0.8058	0.0015
*DFR*	TRINITY_DN42380_c0_g4_i2_2	Dihydroflavonol 4-reductase LrDFR2	0.8045	0.0016
	TRINITY_DN43960_c0_g1_i7_3	Dihydroflavonol 4-reductase (LrDFR1)	0.8655	0.0002
Alfin-like	TRINITY_DN45802_c0_g1_i2_1	PHD finger protein ALFIN-LIKE 6-like	−0.9302	1.16E-05
AP2/ERF	TRINITY_DN42881_c0_g1_i1_3	AP2 domain-containing transcription factor 2	0.8584	0.0003
	TRINITY_DN13573_c0_g1_i1_1	AP2 domain-containing transcription factor 2	−0.8304	0.0008
bHLH	TRINITY_DN36174_c0_g1_i1_1	Transcription factor bHLH30-like	−0.8631	0.0002
	TRINITY_DN41224_c0_g1_i1_1	Transcription factor bHLH57-like	−0.8494	0.0004
	TRINITY_DN48856_c0_g1_i6_1	Transcription factor bHLH48-like	0.8305	0.0008
bZIP	TRINITY_DN27549_c0_g1_i3_2	bZIP transcription factor 11-like	0.8317	0.0007
GATA	TRINITY_DN30983_c0_g2_i1_2	GATA transcription factor 3-like isoform X2	−0.8175	0.0011
GRF	TRINITY_DN37111_c0_g1_i4_2	Growth-regulating factor 4-like	−0.8349	0.0007
	TRINITY_DN48073_c0_g1_i1_1	Growth-regulating factor 7-like	−0.8084	0.0014
C2H2	TRINITY_DN44933_c0_g1_i2_1	Histone deacetylase HDT2-like	−0.8566	0.0003
	TRINITY_DN48967_c0_g1_i23_1	Histone deacetylase HDT2	−0.8434	0.0005
C3H	TRINITY_DN36142_c1_g2_i3_3	Zinc finger CCCH domain-containing protein 8	−0.8517	0.0004
	TRINITY_DN42291_c0_g1_i1_1	Zinc finger CCCH domain-containing protein 59	−0.8752	0.0001
	TRINITY_DN60484_c0_g1_i1_1	Zinc finger CCCH domain-containing protein 44	−0.8130	0.0013
	TRINITY_DN48680_c0_g1_i6_1	Zinc finger CCCH domain-containing protein 8	−0.8535	0.0004
MADS	TRINITY_DN50153_c1_g1_i10_1	Transcription factor, MADS-box	−0.8239	0.0009
MYB	TRINITY_DN33872_c0_g1_i1_3	MYB transcription factor	0.8101	0.0013
	TRINITY_DN45447_c0_g1_i7_1	MYB transcription factor	−0.8298	0.0008
	TRINITY_DN51496_c0_g1_i9_1	MYB transcription factor	−0.8245	0.0009
NAC	TRINITY_DN39353_c0_g2_i1_1	NAC domain-containing protein 43	−0.8351	0.0007
	TRINITY_DN39797_c0_g2_i6_3	NAC domain-containing protein 17-like	0.8215	0.0010
NF-X1	TRINITY_DN33064_c0_g1_i1_2	NF-X1-type zinc finger protein NFXL2	0.8369	0.0006
Trihelix	TRINITY_DN39907_c1_g1_i5_1	Trihelix transcription factor GTL1	0.8138	0.0012
	TRINITY_DN41155_c0_g2_i2_2	Trihelix transcription factor GTL1-like	0.8208	0.0010
	TRINITY_DN43481_c0_g2_i1_1	Trihelix transcription factor ASIL2-like	0.8571	0.0003
	TRINITY_DN45677_c0_g1_i7_3	Trihelix transcription factor GTL1	0.8042	0.0016

### Identification of Transcription Factors Related to Anthocyanin Biosynthesis in the Petals of *Lycoris radiata*

**FIGURE 5:**
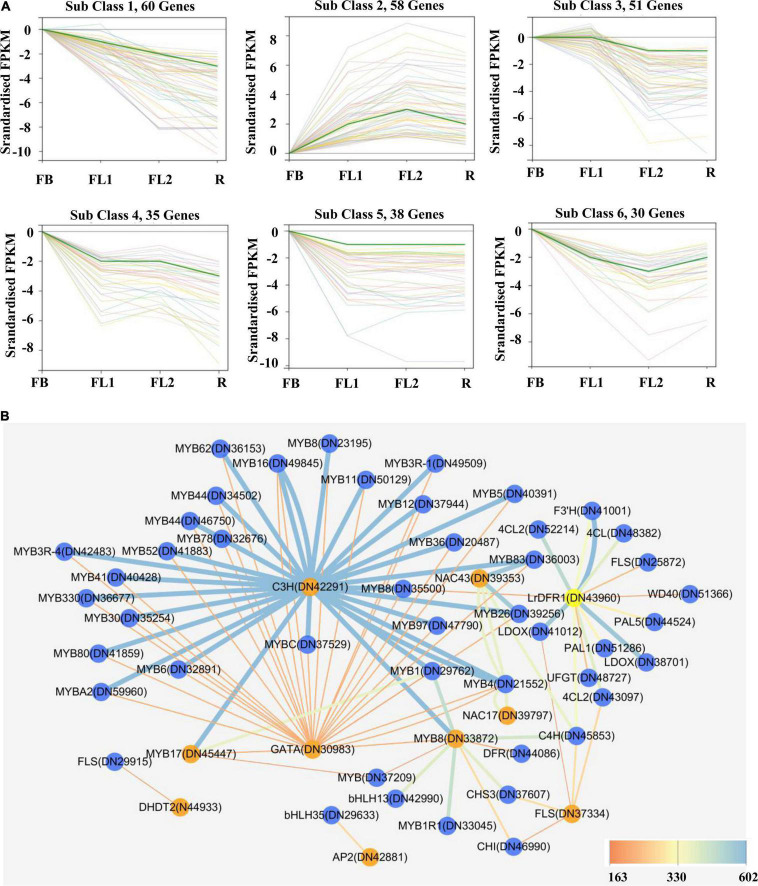
Gene expression profiles of identified transcription factor and protein-protein interaction network of key structural enzymes and TFs involved in anthocyanin biosynthesis in *L. radiata* flowers. **(A)** *K*-means clusters of DEGs based on standardized (log_2_ transformed) FPKM of *L. radiata* petals at flower development stage (FB, FL1, FL2, and R). Number of genes that were clustered in every subclass are shown above each Figure. **(B)** Protein–protein interaction network constituted by protein sequences of differentially expressed transcription factors and structural genes involved in anthocyanin synthesis of *L. radiata* petals. Genes that have the higher weight are depicted in ‘yellow and orange,’ the ‘blue edges’ correspond to co-expressed strong links and the ‘yellow edges’ correspond to co-expressed weak links.

Transcription factors were subsequently predicted to whether modulate anthocyanin accumulation and biosynthesis in *L. radiata* petals during flowering development stages. In this study, a total of 1,631 TFs were identified by searching the TF database. The classified results indicated that most of these TFs belonged to the MYB, C2C2, AP2/ERF, C2H2, and bHLH family (Supplementary Figure 9). Furthermore, the differentially expressed TFs (721) were characterized by analyzing their FPKM values (Supplementary Figure 9). Importantly, co-expression modules of these 721 TF DEGs were analyzed with Short Time-series Expression Miner (STEM) software. In all, six clusters of 272 TF DEGs were detected (Figure 5A and Supplementary Table 8).

On the other hand, by calculating the PCC between the expression level of 721 TF DEGs and the total anthocyanins content, 27 TFs genes (|PCC| > 0.8) involved in the accumulation of anthocyanins were identified, including 10 positive regulators and 17 negative regulators (Table 1). These 10 positive regulators, including *MYB* (1), *AP2*/*ERF* (1), *bHLH* (1), *bZIP* (1), *NAC* (1), *NF-X1* (1), and *Trihelixs* (4) genes, likely act to improve anthocyanin synthesis during *L. radiata* petal development stages. However, the 17 negative regulators, including *Alfin*-like (1), *AP2*/*ERF* (1), *GATA* (1), *GRFs* (2), *bHLHs* (2), *MYBs* (2), *C2H2s* (2), *C3Hs* (4), *MADS* (1), and *NAC* (1), might act as repressors in *L. radiata* anthocyanin biosynthesis (Table 1). Notably, 11 of 17 negative TF regulators (subclass 4, 5, and 6) and one positive regulator (subclass 2) were enriched in TF co-expression modules (Supplementary Table 8).

Previous studies have reported that bHLH, MYB and WD40 TFs regulate anthocyanin biosynthesis thereby activating or repressing transcription of anthocyanin structural genes. We then performed unigenes regarding to MYB, bHLH and WD40, as well as 56 DEGs involved in anthocyanin biosynthesis (Figure 5) to analyze their interaction network and hope to identify the hub TF genes that could affect anthocyanin biosynthesis pathway. The results showed that four *DFRs*, four *MYBs*, two *WD40s*, two *4CLs*, one *F3′H*, one *UFGT*, one *CHS*, one *ANS*, one *FLS*, and one *CHI* were selected as hub genes based on their connection position in the network modules, expression pattern and functional annotation (Supplementary Table 9a and Supplementary Figure 10). Furthermore, those genes (shown in Supplementary Table 9a) and 27 key TF genes (Table 1) were selected to build the interaction network for further analysis. Among them, *LrDFR1* (DN43960) and *LrFLS* (DN37334) could be regarded as key hub genes for participating anthocyanin biosynthesis. Two *MYBs* (DN45447 and DN33872), two *NACs* (DN39353 and DN39797), one *C3H* (DN42291), and one *GATA* (DN30983) TF genes were identified as hub genes in regulating anthocyanin biosynthesis (Figure 5B and Supplementary Table 9b). The above results indicate that these eight genes may play essential roles in anthocyanin synthesis in *L. radiata* during petal development.

### Validation of RNA-seq Data by qRT-PCR

**FIGURE 6:**
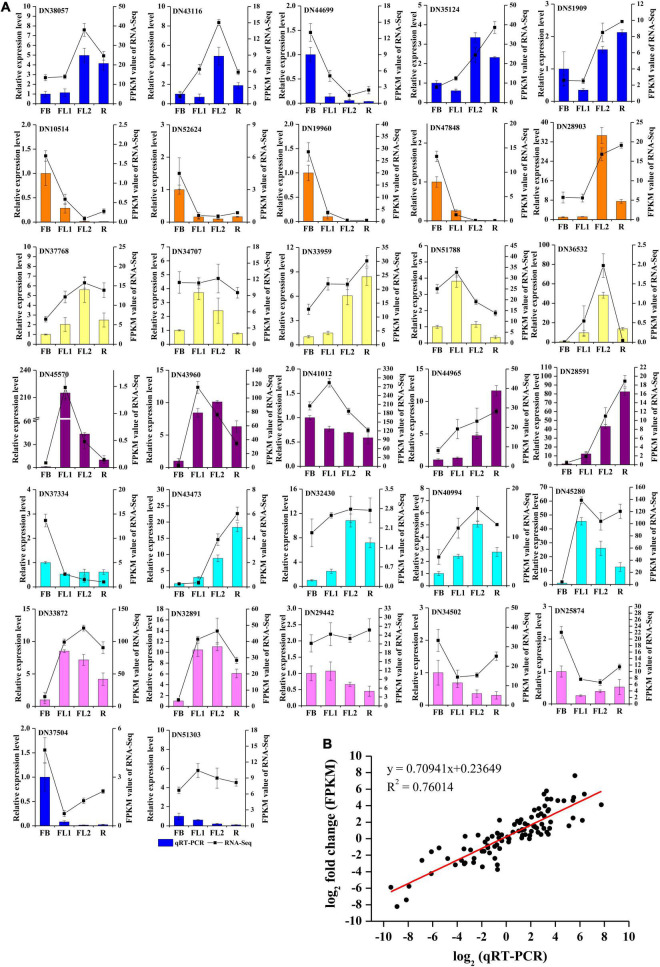
qRT-PCR validation of gene expression level in the transcriptome. **(A)** qRT-PCR validation of gene expression level in the transcriptome. Thirty two unigenes were selected for qRT-PCR validation. **(B)** Correlation analysis of the results between qRT-PCR and RNA-Seq.

To validate the accuracy and transcription profiles revealed by the RNA-seq data, 32 unigenes were selected for qRT-PCR assays. The relative expression levels of these 32 genes were normalized to the expression of *LrTIP41*, and compared with the RNA-Seq data, as shown in Figure 6A. Further linear regression analysis revealed that the expression levels of these genes were well correlated with the RNA-Seq results (Figure 6B, *R*^2^ > 0.76), indicating that the RNA-seq data were credible and accurate.

### *LrDFR1* Is Involved in Anthocyanin Biosynthesis in *Lycoris radiata*

**FIGURE 7:**
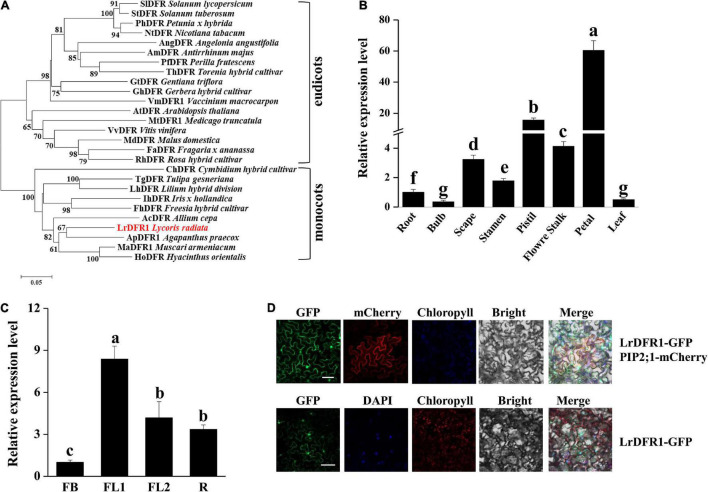
Phylogenetic tree analysis, transcription expression profiles, and subcellular localization of LrDFR1. **(A)** Phylogenetic assessment of LrDFR1 with other DFR proteins from different plants. Phylogenetic tree generation was achieved using the maximum likelihood method in MEGA 7.0 software. Numbers at every interior branch show bootstrap values of 1000 replicates. The bar shows a 0.05 genetic distance. Plant species as well as GenBank accession numbers of DFR proteins used in phylogenetic analyses are: *Solanum tuberosum* StDFR (AF449422), *Solanum lycopersicum* SlDFR (CAA79154.1), *Petunia hybrida* PhDFR (AF233639), *Angelonia angustifolia* AngDFR (KJ817183), *Nicotiana tabacum* NtDFR (NP_001312559.1), *Antirrhinum majus* AmDFR (X15536), *Perilla frutescens* PfDFR (AB002817), *Gentiana triflora* GtDFR (D85185), *Torenia hybrid* ThDFR (AB012924), *Gerbera hybrid* GhDFR (Z17221), *Vaccinium macrocarpon* VmDFR1 (AF483835), *Arabidopsis thaliana* AtDFR (AB033294), *Medicago truncatula* MtDFR1 (AY389346), *Vitis vinifera* VvDFR (Y11749), *Malus domestica* MdDFR (AAO39816), *Rosa hybrid* RhDFR (D85102), *Cymbidium hybrid* ChDFR (AF017451), *Fragaria ananassa* FaDFR (AF029685), *Tulipa gesneriana* TgDFR (BAH98155.1), *Lilium hybrid* LhDFR (AB058641), *Iris hollandica* IhDFR (BAF93856.1), *Allium cepa* AcDFR (AY221250.2), *Agapanthus praecox* ApDFR (AB099529.1), *Muscari aucheri* MaDFR (MH636605), *Freesia hybrid* FhDFR (KU132389), and *Hyacinthus orientalis* HoDFR (AFP58815.1). **(B)** Expression profiles of *LrDFR1* in various tissues of *L. radiata*. Expressions of *LrDFR1* were assessed by qRT-PCR, and normalized to *LrTIP41*. Expressions of *LrDFR1* in root tissues were defined as 1.0. Data are shown as mean ± SD. Bars with different letters are significantly different at *p* < 0.05 according to Duncan’s multiple range test. **(C)** Expression profiles of *LrDFR1* during the FB stage, FL1 stage, FL2 stage and R stage of *L. radiata*. Expression levels of *LrDFR1* were assessed by qRT-PCR, and normalized to *LrTIP41*. Expression levels of *LrDFR1* in FB stage were defined as 1.0. Data are shown as mean ± SD. Bars with different letters are significantly different at *p* < 0.05 according to Duncan’s multiple range test. **(D)** Subcellular localization of LrDFR1 in *N. benthamiana* epidermal cells. Scale bars = 20 μm. The nuclei are indicated by DAPI staining.

In this study, we cloned *LrDFR1* gene (DN43960) from *L. radiata*. The full-length cDNA of *LrDFR1* is 1113 bp in length and it encodes a 370 amino acid protein with a molecular weight of 41.67 kDa (Supplementary Table 10). The deduced amino acid sequence of LrDFR1 revealed a high similarity with DFR proteins from *Agapanthus praecox* (75.33%), *Muscari armeniacum* (74.74%), and *Hyacinthus orientalis* (72.72%) (Figure 7A). Multiple amino acid sequence alignments showed the highly preserved NADPH-binding motif (VTGAAGFIGSWLIMRLLERGY) (Gang, 2005) and the substrate-binding domain (T128–K154) (Johnson et al., 2001) in the LrDFR1 sequence (Supplementary Figure 11). qRT-PCR was then performed to assess whether expression patterns of *LrDFR1* in different tissues and flower development stages were coincided with anthocyanin accumulation in *L. radiata*. *LrDFR1* was found to be expressed in all tissues, with the highest expression levels in petals (Figure 7B). Moreover, expression levels of *LrDFR1* were significantly increased from stage FB to stage R, peaking at stage FL1 (Figure 7C). These findings imply tissue-specific expression levels for *LrDFR1*, which is associated with anthocyanin accumulation in *L. radiata* petals.

**FIGURE 8:**
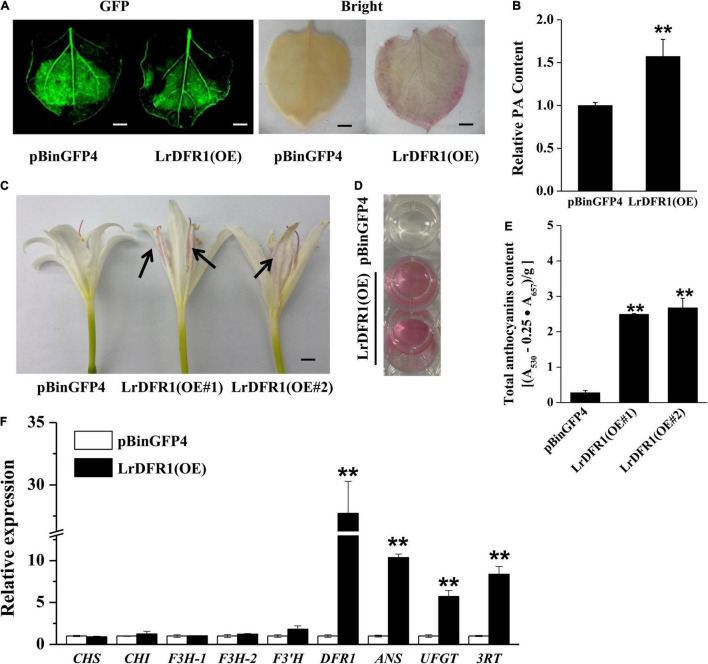
Overexpression of *LrDFR1* promotes anthocyanin and proanthocyanidin biosynthesis. **(A)** Proanthocyanidin staining and **(B)** relative proanthocyanidin (PA) levels in transiently transformed tobacco leaves (pBinGFP4: empty vector controls; LrDFR1-OE: *LrDFR1-*overexpressing leaves). Tobacco leaves were kept in a phytotron at 24°C under constant lighting for 5 days. DMACA was used to stain proanthocyanidin. Every experiment was performed using 8–10 leaves for each genotype. Experiments were conducted in triplicates, and a representative image is shown. Proanthocyanidin levels of empty vector controls were set as the reference to 1. Asterisks represent significant differences between control and *LrDFR1*-overexpressing leaves (**p < 0.01). Bars = 1 cm. **(C)** Phenotypes of anthocyanin accumulation. Arrow indicates the transfected petals. **(D,E)** Relative anthocyanin levels in transiently transformed *Lycoris* petals (pBinGFP4: empty vector controls; LrDFR1-OE: *LrDFR1-*overexpressing petals). *Lycoris* petals were kept in a phytotron at 24°C with a constant light for 5 days. Every experiment was performed using 8–10 petals per genotype. Data are shown as mean ± SD. **p < 0.01. Bar = 0.5 cm. **(F)** Relative expression levels of endogenous anthocyanin biosynthetic genes in pBinGFP4 (empty vector controls) as well as *LrDFR1*-overexpressing petals. Expression patterns of early biosynthetic genes (*CHS*, *F3H*, *CHI*, and *F3′H*) as well as late biosynthetic genes (*DFR*, *UFGT, ANS*, and 3RT) in petals were investigated. Asterisks represent significant differences between control and *LrDFR1*-overexpressing petals (**p < 0.01).

Moreover, we transiently expressed *LrDFR1* in tobacco epidermal cells to assess subcellular localization of *LrDFR1*. As shown in Figure 7D, the fluorescent signal of LrDFR1-GFP was localized into the nucleus, cytoplasm and cell membrane, while GFP was evenly distributed in the cell (Figure 7D and Supplementary Figure 12). To determine the roles of *LrDFR1* in regulating anthocyanin as well as proanthocyanidin biosynthesis in *L. radiata*, an *LrDFR1*-overexpressing plasmid was transfected into *Lycoris* petals and tobacco epidermal cells (Figure 8A). Overexpression of *LrDFR1* in tobacco and *Lycoris* petals markedly enhanced proanthocyanidin and anthocyanin accumulation (Figures 8B–E). To assess the effects of *LrDFR1* on endogenous *Lycoris* petals genes that are involved in anthocyanin synthesis, the expression levels of *CHS*, *CHI*, *F3H*, *F3′H*, *DFR*, *ANS*, *UFGT*, and *3RT* were determined (Figure 8F). Among them, the expressions of *LrDFR1*, *ANS*, *UFGT* and *3RT* were significantly higher in *LrDFR1-*overexpressing plants than in control plants (Figure 8F). These results suggest that *LrDFR1* may play important roles in anthocyanins biosynthesis of *Lycoris* petals.

## Discussion

### Changes of Anthocyanin Contents in the *Lycoris radiata* Petals During Flower Development Stages

Flowering plants exhibit a wide variation in their flora, foliage, and fruit colors, as a result of genetic factors and variations in environments. Flavonoids/anthocyanins, betalains and carotenoids are the major metabolites for coloration in plant reproductive organs (Griesbach, 2005; Tanaka et al., 2008). Most of the red, purple, and blue-colored flowers (such as red rose, lavender, and blue chicory) as well as fruits (such as berries, currants, and grapes) contained high anthocyanins content (Khoo et al., 2017). The genus *Lycoris* is used as a garden flower due to the colorful and special flowers, and the flower colors of *Lycoris* are diverse. For example, the flower color of *L. radiata* and *L. rosea* was red, that of *L. aurea* and *L. chinensis* was yellow. *L. sprengeri* and *L. haywardii* showed red and blue color, while *L. longituba* displays an exceptionally wide diversity of flower colors from purple, red, orange, to yellow (He et al., 2011). Similar to the flowers of other species, the petals of *Lycoris* are rich in anthocyanins, and their color formation are largely related to anthocyanins (He et al., 2011; Chun et al., 2013; Yue et al., 2019; Park et al., 2021). In this study, we determined the content of anthocyanins in the petals of *L. radiata*, and the results showed that the color intensity of the *L. radiata* petals was changed with the different anthocyanin contents. The anthocyanins increased then decreased during the flower development stages (Figure 1), which are similar to the results recently reported by Park et al. (2021).

### Key Structural Genes Responsible for Anthocyanin Synthesis in *Lycoris radiata* Petals During Flower Development Stages

To data, transcriptome sequencing is highly employed for predicting novel genes, gene function, and genome evolution for plant breeding and horticulture research (Rameneni et al., 2020). For example, transcriptome analysis has revealed the role of anthocyanin in flower color formation in several horticultural crops, such as *Camellia sinensis* (Zhou et al., 2020), “Tiny Padhye” (*Lilium* spp.) (Xu et al., 2017), lilies (*Lilium* spp.) (Suzuki et al., 2016), *Magnolia sprengeri* (Shi et al., 2014), *Paeonia lactiflora* (Zhao et al., 2014), *Paeonia delavayi* (Shi et al., 2015), and *Silene littorea* (Casimiro-Soriguer et al., 2016). For better understanding of petals color formation during flower development stages in *L. radiata*, a comparative transcriptomics analysis was carried out. The results showed that approximately 70.27 GB of high-quality data, and 87,584 unigenes were obtained. Further analyses, based on NR, Swiss-Prot, KEGG, KOG, GO, Pfam, and eggNOG databases, predicted 38,798 DEGs associated with a specific or general function (Supplementary Tables 4, 5).

The variations in floral coloration emanates from different processes, such as pathways competition, expression levels of structural genes involved in pigment formation, and mutations of structural or regulatory genes (Grotewold, 2006; Cui et al., 2021). In plants, phenylpropanoids represent a vital group of physiologically active secondary metabolites derived from phenylalanine, and anthocyanins, flavonols, isoflaconoids and flavonols have a similar metabolism pathway during their biosynthesis (Ferrer et al., 2008). KEGG pathway analysis showed that the ‘phenylpropanoid biosynthesis,’ ‘flavonoid biosynthesis,’ as well as ‘flavone and flavonol biosynthesis’ pathways were enriched between each two transcriptomes of *L. radiata* petals during flower development stages (Figure 3B and Supplementary Figure 8). Given that anthocyanin biosynthesis pathway is well known to modulate color formation in plants, we mainly focused on them as the candidate pathways to elucidate their involvement in petal/flower color formation in *L. radiata*. Subsequently, we identified the main functional genes participated in the anthocyanin biosynthetic pathway, and found that most of structural genes such as *F3′H*, *UFGT*, *DFR*, and *FLS* were elevated in *L. radiata* petals at FL1 and FL2 stages (Figure 4A). Therefore, these genes might have contributed to the increasing anthocyanin content in petals from the FB stage to the FL1 and FL2 stage, as evidenced in Figure 1. For example, three *F3′H* genes (*DN41001*, *DN43758*, and *DN46768*) were highly expressed in petals at FL1 and FL2 stages (Figure 4A). Another prominent gene, *UFGT* (*DN44965*), which glycolyzes anthocyanidin into anthocyanin (Xie et al., 2003), was also highly expressed in petals at FL1, FL2 and R stages, as compared to that of the samples at FB stage (Figure 4A). All of these genes were positively correlated with the biosynthesis of anthocyanins (Niu et al., 2010). Notably, two *DFR* genes (DN42380, DN43960) and one *FLS* (DN37334) (Table 1) were found to be highly associated with the total anthocyanins content (|PCC| > 0.8), suggesting they may have an essential function in the phenotypic expression of petal color (Figure 4B). In anthocyanin biosynthesis, DFR catalyze the reduction of dihydroquercetin to leucoanthocyanidins, and the level of *DFR* expression have been associated with flower color changes (Nakatsuka et al., 2003; Zhao et al., 2012). qRT-PCR also indicated that the hub gene *LrDFR1* were mostly expressed the most in the FL1 samples (Figure 7C). Our results suggest that these enzymes may be the most important enzymes to catalyze anthocyanin biosynthesis in *L. radiata* petals.

### Transcriptional Regulation of Color Formation in *Lycoris radiata* Petals

Transcription factors play critical functions in flavonoid biosynthesis, by regulating expression of structural genes. For example, the class of TFs identified were previously implicated in regulation of petal color formation in roses (Li D. et al., 2020). Particularly, MYB-bHLH-WD40 complexes have been implicated in multi-level regulation of flavonoid biosynthesis (Gallego et al., 2018), whereas the R2R3-MYB family was shown to play a vital role in regulation of spatiotemporal expressions of genes involved in anthocyanin biosynthetic in plants (Gonzalez et al., 2008; Zhao and Tao, 2015). Besides, MYB-domain TFs are important mediators of anthocyanin accumulation and participate in colorations of various organs in horticultural as well as ornamental plants (Tang et al., 2017; Liu Y. et al., 2019; Xiang et al., 2019; Zhai et al., 2019; He et al., 2020; Sun et al., 2020; Wang et al., 2020; Zhong et al., 2020).

In this study, the most abundant TFs including *AP2*/*ERF*, *bHLH*, *bZIP*, *C2C2*, *HSF*, *MYB*, *NAC*, *TIFY*, and *WRKY* families were predicted (Supplementary Table 8). In addition, we employed a *K*-means clustering, as proposed earlier by Handhayani and Hiryanto (2015), which permitted the clustering of 272 TF unique genes among the samples (FB, FL1, FL2, and R) into six sub-clusters with some members in Cluster 2 associated with genes from the *MYB* and *bHLH* TFs (Figure 5A). Based on the expression level of TFs obtained from the transcriptome data, 27 TFs (Table 1) were found to highly associate with the total anthocyanin content (|PCC| > 0.8), and these TFs may have an essential function in the phenotypic expression of *L. radiata* petal color. Interestingly, among these TFs, three *MYBs* showed two different expression patterns. The expression level of two *MYBs* (DN45447, DN51496) was highest in FB, followed by FL1, FL2, and R, which was contrary to the total anthocyanin content trend. Conversely, the expression of *LrMYB1* (DN33872) exhibited a similar trend to the total anthocyanin content in the *L. radiata* petals (Supplementary Figure 13), indicating that *MYBs* (DN45447 and DN51496) negatively regulated anthocyanin accumulation, whereas *LrMYB1* (DN33872) was identified as one of the eight hub genes may positively regulate anthocyanin accumulation in *L. radiata* (Table 1 and Figure 5B).

Subsequently, two negatively correlated *bHLHs* (DN36174 and DN41224) and one positively correlated *LrbHLH1* (DN48856) were identified (Table 1). In plants, MYB often forms protein complexes with bHLH and WD40 to participate in anthocyanin biosynthesis rather than regulate anthocyanin biosynthesis directly (Feng et al., 2020). In apple, *MdMYB1*, *MdMYB9*, *MdMYB10*, and *MdMYBA* act as positive modulators of anthocyanin biosynthesis, by activating the expressions of *MdDFR* and *MdUF3GT* (Takos et al., 2006; Ban et al., 2007; Espley et al., 2007; An et al., 2015). On the contrary, downregulation of *MdMYB1* inhibits anthocyanin accumulation mediated by ethylene, abscisic acid (ABA), wounding, drought, and different light intensities (An et al., 2018, 2019, 2020a,b). Notably, our results also revealed a significant upregulation of *LrMYB1* (DN33872) and *LrbHLH1* (DN48856), of which the expression was positively correlated with *LrDFR1, LrCHS*, *LrCHI*, *F3′H, LrUFGT* and *LrANS* genes during petal development stages (Supplementary Figure 13). This is similar to that of *LhMYB12*-Lat, which has previously been associated with activation of accumulation of anthocyanin in lily petals (Yamagishi et al., 2014). In our co-expression networks, the module that was positively correlated with anthocyanin contents and modules negatively correlated with anthocyanin content were identified. Overall, whether these *MYB* TFs interact with *bHLH* TFs to regulate anthocyanin biosynthesis in *L. radiata* remains to be further investigated.

### The *LrDFR1* Drives Anthocyanin Accumulation in *Lycoris radiata* Petals

In the anthocyanin biosynthesis pathway, *DFR* catalyzes dihydroflavonol conversion to leucoanthocyanidins (Zhang et al., 2014). *DFR* belongs to the superfamily of short chain dehydrogenase reductase (SDR), which has a highly preserved NADPH-binding domain “VTGAAGFIGSWLIMRLLERGY” as well as a substrate-binding domain in plants (Martens et al., 2002; Haselmair-Gosch et al., 2018). In this study, based on the expression level of the anthocyanin structure genes obtained from the transcriptome data, *LrDFR1* and *LrDFR2* (Table 1) were found to highly associate with the total anthocyanin content (PCC > 0.8), suggesting *DFR* may have an essential function in the phenotypic expression of *L. radiata* petal color. *LrDFR1* was then identified as one of the hub genes (Figure 5B) and important to positively regulate anthocyanin production in *L. radiata* petals. Multiple amino acid alignments showed that LrDFR1 contains the NADPH-binding domains and substrate-binding domains. Phylogenic tree analysis revealed a high similarity between LrDFR1 and other characterized DFRs, implying that LrDFR1 belongs to the monocot DFR family and exhibits catalytic characteristics.

The *DFR* genes of *Iris* and *Gentiana* have been reported to be associated with the absence of brick-red flowers (Noda et al., 2017). Moreover, heterologous *MaDFR* expressions in *N. tabacum* has been associated with enhanced anthocyanin accumulation, which leads to darker flower colors, suggesting that *MaDFR* is involved in flower color development (Liu H. et al., 2019). After the introduction of maize (*Zea mays*) *DFR* into white-flowered petunia varieties, transgenic plant flowers accumulate non-native pelargonidin, which results in novel brick red-flower varieties (Meyer et al., 1987). In this study, the expression patterns of *LrDFR1* was first temporally and spatially tested in various tissues and petal development stages of *L. radiata*. It showed that the expression levels of *LrDFR1* were correlated with total anthocyanin accumulation. These findings imply that *LrDFR1* is associated with petal color development in *L. radiata* (Figures 1, 7C,D). The spatial and temporal expression characteristics of *LrDFR1* gene were found similarly in several other species (Liu H. et al., 2019; Lim et al., 2020). In order to investigate the functional divergence of *LrDFR1* gene in the flavonoid biosynthesis, we performed transient expression analyses using *Lycoris* petals and tobacco leaves. Overexpressed *LrDFR1* was associated with significantly elevated anthocyanin content and proanthocyanidin content in *Lycoris* petals and tobacco leaves. Interestingly, overexpression of *LrDFR1* also enhanced the expression of downstream genes (*LrANS* and *LrUFGT*) involved in anthocyanins biosynthesis in transgenic *Lycoris* petals (Figures 8B–F). In addition, for plant breeders, a single *DFR* gene maybe ideal for determining flower colors. *DFR* is vital for pigmentation, when compared to other anthocyanin biosynthetic genes, which only regulate plant flower color hue. Thus, whether *LrDFR1* has a high preference for dihydromyricetin, and is accountable for the limited flower colors in *L. radiata* needs further study.

## Conclusion

In this study, we provided a dynamic transcriptome profile of *L. radiata* petals during flower development stages. Overall, 56 structural genes and 27 key TF DEGs were identified as key genes responsible for *L. radiata* petal coloration. In the protein-protein interaction network analysis, *LrDFR1* was identified as a hub gene in the anthocyanin biosynthesis pathway, and was highly associated with anthocyanin accumulation. Overexpression of *LrDFR1* in *Lycoris* petals and tobacco leaves induced anthocyanin accumulation. In addition, the structural genes and co-expressed TFs reported in this study would serve as useful genetic resources for further functional characterization and molecular breeding programs in *L. radiata.* Taken together, our results elucidate on the molecular basis of petal development in *L. radiata*.

## Data Availability Statement

The datasets presented in this study can be found in online repositories. The names of the repository/repositories and accession number(s) can be found below: https://ngdc.cncb.ac.cn/, CRA004779.

## Author Contributions

NW and ZW designed the research and wrote the manuscript. NW performed most of the experiments and data analysis. XS and FZ collected the experimental materials. TW assisted with the data analysis. WZ provided helpful comments on the manuscript. ZW provided guidance on the whole study and contributed with valuable discussions. All authors read and approved the final manuscript.

## Conflict of Interest

The authors declare that the research was conducted in the absence of any commercial or financial relationships that could be construed as a potential conflict of interest.

## Publisher’s Note

All claims expressed in this article are solely those of the authors and do not necessarily represent those of their affiliated organizations, or those of the publisher, the editors and the reviewers. Any product that may be evaluated in this article, or claim that may be made by its manufacturer, is not guaranteed or endorsed by the publisher.
